# Stem Cell-Derived Extracellular Vesicles Ameliorate the Neuron Mitochondrial Damage Induced by ROS-, LPS-Exposure: In Vitro Model of Neuron, Microglia, and Astrocyte Triple Co-Culture

**DOI:** 10.3390/ijms27114834

**Published:** 2026-05-27

**Authors:** Marta Malenchini, Francesca Beretti, Martina Gatti, Emma Bertucci, Elena Del Toro, Tullia Maraldi

**Affiliations:** 1 Department of Biomedical, Metabolic and Neural Sciences, University of Modena and Reggio Emilia, 41125 Modena, Italy; marta.malenchini@unimore.it (M.M.); francesca.beretti@unimore.it (F.B.); or martina.gatti6@unibo.it (M.G.); 290498@studenti.unimore.it (E.D.T.); 2 Cellular Signalling Laboratory, Department of Biomedical and Neuromotor Science, University of Bologna, 40125 Bologna, Italy; 3 Department of Medical and Surgical Sciences for Mothers, Children and Adults, University of Modena and Reggio Emilia, 41124 Modena, Italy; emma.bertucci@unimore.it

**Keywords:** neurons, LPS, ROS, mitochondria, extracellular vesicles

## Abstract

Oxidative stress causes brain damage contributing to neurodegenerative and vascular diseases. In Alzheimer’s disease (AD), elevated oxidative stress and mitochondrial damage are closely linked to misfolded protein accumulation. ROS also plays a major role in ischemic brain injury, particularly during reperfusion, impairing the blood–brain barrier and highlighting the association between vascular pathology and AD. To investigate perturbations in brain cells occurring in mixed dementia (AD combined with vascular dementia components), we used a triple culture system comprising neurons, astrocytes, and microglia and induced neuronal injury by combining LPS and H_2_O_2_ exposures. Cell viability assays revealed that neuronal death occurred mainly through apoptosis and DNA damage. In neurons and astrocytes exposed to LPS+H_2_O_2_, the expression of NADPH oxidase isoform 2, a major source of ROS, increased, along with FOXO3 and SOD2, a key mitochondrial ROS scavenger. Indeed, these changes were accompanied by altered mitochondrial morphology and integrity, as well as reduced neurite extension and thickness. The treatment with extracellular vesicles (EVs) derived from amniotic fluid stem cells was tested due to their rich content of antioxidant molecules. Interestingly, EVs reversed the negative effects of LPS+H_2_O_2_, suggesting the protective role against neuronal injury in vitro may be mediated by the EV-cargo.

## 1. Introduction

Mixed dementia, defined by the co-occurrence of Alzheimer’s disease (AD) and vascular dementia (VaD), represents one of the most prevalent and challenging forms of cognitive decline in the aging population. Patients with mixed pathology often display faster cognitive deterioration, more severe behavioral disturbances, and poorer prognosis compared to individuals with a single type of dementia [1]. Oxidative stress has been increasingly recognized as a central and convergent mechanism in mixed dementia, linking vascular pathology, amyloid deposition, tau hyperphosphorylation, and neuroinflammation [2,3]. Oxidative stress emerges as a unifying pathogenic mechanism in mixed dementia, and advanced multicellular in vitro models could provide a unique opportunity to investigate its effects in a physiologically relevant context.

The complex interplay among neurons, astrocytes, and microglia is central to both the propagation of oxidative damage and the regulation of neuroinflammatory responses [4]. Consequently, in vitro co-culture systems that integrate these three cell types have emerged as essential tools to dissect cellular and molecular mechanisms in mixed dementia. Tri-culture models allow the simulation of physiologically relevant cell–cell interactions [5], either through direct physical contact or via the secretome, and enable the investigation of oxidative stress dynamics and assessment of neuroprotective or anti-inflammatory interventions under controlled experimental conditions.

Traditional 2D cultures often fail to recapitulate the spatial organization and microenvironment of the brain, whereas 3D models, including spheroids and organoids, promote more realistic cellular architecture, intercellular signaling, and metabolic gradients. However, these complex systems show some limits in the study of events occurring in each cell type through western blot or RT-PCR analysis, such as extraction difficulties, lower yield, higher variability, matrix interference, and especially loss of spatial information due to the intrinsic heterogeneity of 3D systems. Indeed, in mixed-cell spheroids, organoids, or scaffold-based co-cultures, proteins and RNA extracted from the entire construct are typically derived from all cellular populations simultaneously. Consequently, bulk lysates obscure the relative contribution of each cell type [6]. To overcome this limitation, we preferred the use of a transwell system to obtain a triple cell-culture brain model.

Our aim was to use this system to develop a mimetic in vitro model of mixed dementia. To mimic AD, these cells may be treated with Aβ oligomers, tau aggregates, or bacterial endotoxin lipopolysaccharide (LPS). We used LPS since several studies suggest that AD might be a neurological disorder associated with infectious agents and that different bacterial infections could trigger downstream AD pathology [7]. To model VaD, cells can be subjected to oxygen/glucose deprivation, hypoxia-reoxygenation, or a direct hydrogen peroxide (H_2_O_2_) treatment [8], as we selected in this study. Indeed, H_2_O_2_ treatment of cultured neuronal cells is commonly used to mimic oxidative stress in vitro [9], which arises from cerebrovascular damage and ischemia–reperfusion injury.

Our purpose was to recreate a scenario in which ischemic events, causing oxidative stress, disrupt the blood–brain barrier, allowing entry of LPS. LPS exposure may then favor H_2_O_2_ uptake by neurons and astrocytes and activate microglia, creating a vicious circle that promotes protein aggregation and cellular damage. Although this is a simplified model that cannot recapitulate the disease completely, the use of LPS + H_2_O_2_ was intended to model the upstream convergence of inflammatory and oxidative mechanisms characteristic of mixed dementia, thereby mimicking disease initiation and progression more closely than exogenous Aβ oligomers, which primarily reproduce an already established amyloid-toxic phenotype.

Thus, neurons, astrocytes, and microglia were exposed to co-treatment with H_2_O_2_ and LPS in order to evaluate their responses to these insults using parameters such as cell viability, activation status, cytokine release, and ROS production [1].

Stem cell-derived extracellular vesicles (EVs), including exosomes and microvesicles, have garnered attention as innovative therapeutic agents for neurodegenerative diseases. EVs contain a complex cargo of proteins, lipids, and nucleic acids capable of modulating recipient cell behavior, reducing oxidative stress, and promoting cellular survival [10]. Recent studies have demonstrated that EVs derived from mesenchymal stem cells or induced pluripotent stem cells can attenuate ROS-induced damage, modulate glial activation, and enhance neuronal resilience in in vitro models that incorporate neurons, astrocytes, and microglia [11]. Our group previously demonstrated that EVs derived from amniotic fluid stem cells (AFSC-EVs) exhibit antioxidant properties, as they contain superoxide dismutase (SOD) enzymes [12]. Moreover, EV-based therapies may offer a cell-free alternative to direct stem cell transplantation, with the potential to overcome limitations related to immunogenicity, tumorigenicity, and ethical concerns.

Overall, this study aims to investigate how oxidative stress is involved in mixed dementia, highlight the utility of neuron–astrocyte–microglia in vitro models, and examine current evidence supporting EV-based therapies as innovative strategies for disease modification. Furthermore, stem cell-derived EVs represent a promising avenue for therapeutic intervention, offering neuroprotection and modulation of glial responses in complex disease-relevant models.

## 2. Results

### 2.1. Neurotoxicity Induction in the Mono- and Triple-Culture Model

**Figure 1:**
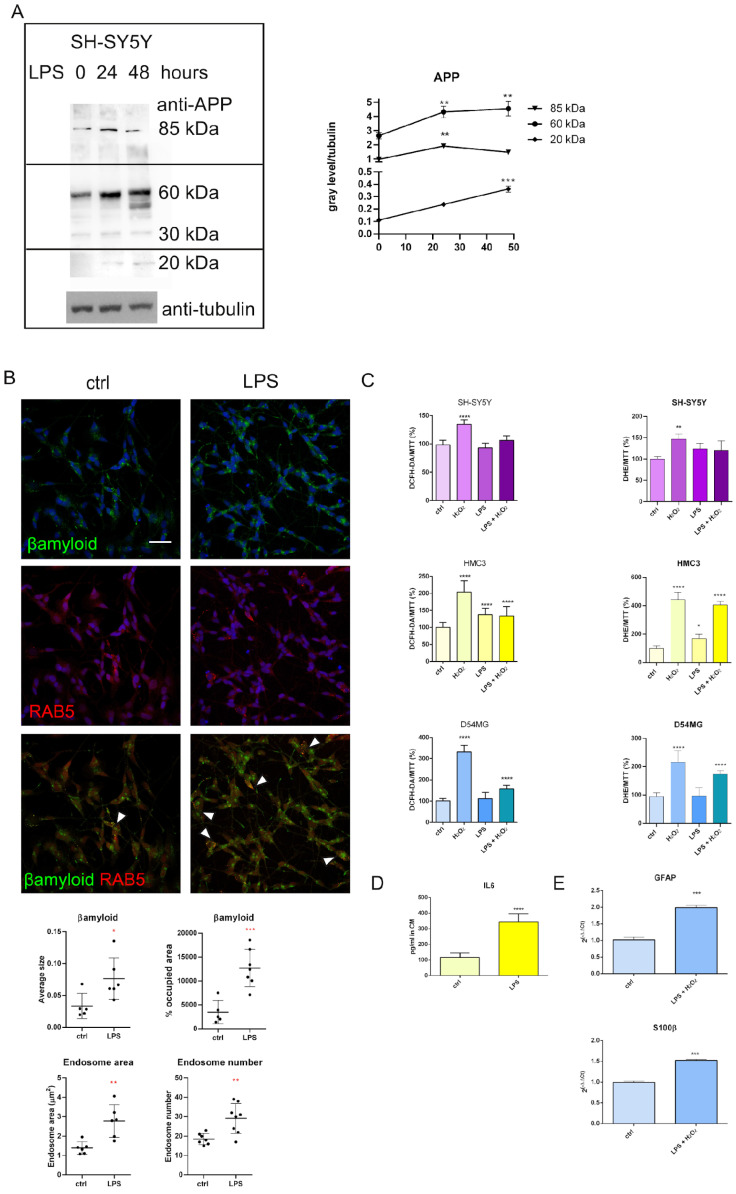
Effects of LPS and H_2_O_2_ treatments on neurons, astrocytes, and microglia cells. (**A**) Western blot analysis of lysates of SH-SY5Y cells, treated with LPS 1 µg/mL for 24 or 48 h, revealed with anti-amyloid precursor protein clone 6E10 (APP) and anti-tubulin, as loading control. Molecular weights where the band intensity changes are indicated. Relative densitometric analysis was reported in the graph. Values were reported as mean (*n* = 3) ± SD: ** *p* value < 0.01, *** *p* value < 0.001. (**B**) Immunofluorescence images of SH-SY5Y cells, treated or not with LPS 1 µg/mL for 48 h, stained with DAPI (blue), anti-APP (green), and anti-RAB5 (red). Arrowheads indicate where the red signal superimposes with the green one. The upper graph represents the average size and the percentage of occupied area of amyloid-β clusters, and the lower shows the dimensions and number of endosomes stained with RAB5. Scale bar: 10 μm. Values were reported as dot plot + mean ± SD: * *p* value < 0.05, ** *p* value < 0.01, *** *p* value < 0.001. (**C**) SH-SY5Y and D54MG were treated with LPS for 48 h and HMC3 for 24 h, all of them were treated with 250 μM H_2_O_2_ for 3 h or in combination (LPS + H_2_O_2_). Graphs represent the intracellular ROS values, evaluated with DCFH-DA and DHE probes, normalized to viability values, and analyzed with MTT test. Values were reported as mean (*n* = 3) ± SD: * *p* value < 0.05, ** *p* value < 0.01, **** *p* value < 0.0001. (**D**) Graph shows the results of ELISA test for IL6 analyzed in the conditioned medium of HMC3 collected after LPS treatment. Values were reported as mean (*n* = 3) ± SD: **** *p* value < 0.0001. (**E**) RT-PCR analysis of expression of S100β and GFAP in D54MG cells after LPS + H_2_O_2_ treatment. Values were reported as mean (*n* = 3) ± SEM: *** *p* value < 0.05.

Here, we used SH-SY5Y, a human neuroblastoma cell line differentiated with retinoic acid (RA), as an in vitro neuronal model. First, the formation of AD-like changes in neurons, such as amyloid-β oligomers, was induced by LPS-treatment: we investigated the modulation of intracellular AD markers using the antibody 6E10, which is specific for human amyloid precursor protein (APP), Aβ oligomers [13], and molecules generated from cleavage of APP by secretases [14]. Immunoblotting results demonstrated that levels of APP and Aβ oligomers between 60 and 20 kDa were enhanced in LPS-treated neurons (Figure 1A). Immunofluorescence analysis confirmed these findings (Figure 1B), as 6E10 staining showed an increase in amyloid-β cluster size and area within neurons that colocalized with endosomes marked by RAB5, following LPS exposure. Indeed, Aβ is generated predominantly in endosomes. After internalization from the plasma membrane, APP traffics to early endosomes, where the acidic environment favors its cleavage by BACE1 (β-secretase), followed by γ-secretase processing, producing Aβ peptides. Therefore, any condition that increases APP internalization, prolongs residence time in endosomes, or impairs endosome maturation can enhance amyloidogenic processing [15,16]. LPS promotes such disturbances.

Then, we demonstrated that the microglial cell line HMC3, treated with LPS, displayed an increase in reactive oxygen and nitrogen species (RONS) production, as expected, unlike neurons and astrocytes (D54MG cell line) (Figure 1C). Here, we used DCFH-DA (dichloro-dihydro-fluorescein diacetate) and DHE (dihydroethidium), which are both membrane-permeable probes retained within the cell, enabling monitoring of intracellular oxidative changes via fluorescence [17]: DHE reacts particularly with superoxide, while DCFH-DA can give overall information on intracellular oxidative stress, not directly reacting with many RONS. The elevated IL6 concentration in the conditioned medium (CM) likely reflects microglial activation (Figure 1D). Astrocyte activation under these conditions was confirmed by RT-PCR analysis for the markers S-100β and GFAP, which are commonly upregulated during reactive astrogliosis following CNS injury or disease (Figure 1E). H_2_O_2_ treatment determined a rise in ROS content in all the cytotypes, although this effect was particularly pronounced in astrocytes, while the co-treatment (LPS + H_2_O_2_) did not potentiate the effect of H_2_O_2_ alone (Figure 1C).

**Figure 2:**
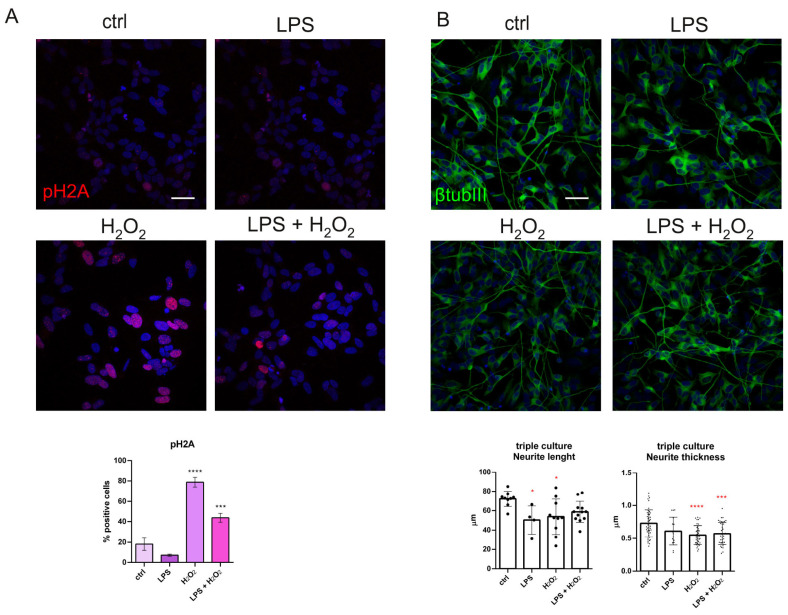
Effects of LPS and H_2_O_2_ treatments on the cell damage of neurons, astrocytes, and microglia cells. (**A**) The graph represents the analysis of immunofluorescence images of SH-SY5Y cells, treated or not with LPS, H_2_O_2_, or LPS + H_2_O_2_ stained with DAPI (blue) and anti-pH2A (red). Scale bar: 10 μm. Values were reported as mean (*n* = 3 images, each showing around 50 cells) ± SD: *** *p* value < 0.001; **** *p* value < 0.0001. (**B**) Immunofluorescence images of SH-SY5Y cells, treated or not with LPS, H_2_O_2_, or LPS + H_2_O_2_ stained with DAPI (blue) and anti-βtubulin III (green). The graphs represent the length and the thickness of neurites. Scale bar: 10 μm. Values were reported as dot plot + mean ± SD: * *p* value < 0.05, *** *p* value < 0.001; **** *p* value < 0.0001.

However, LPS induced cell death in SH-SY5Y cultured alone, and, once in co-culture, cytotoxicity was enhanced (Table 1), while cell viability of microglia and astrocytes was not affected. The acute H_2_O_2_ exposure deeply affected cell viability, even not showing an additive effect with LPS. Phosphorylated Histone H2A.X (H2AX) is commonly employed to detect neuronal DNA damage in neurodegeneration and injury models. Only H_2_O_2_ caused direct DNA damage in neurons (Figure 2A), but all the conditions provoked a decrease in neurite length and thickness (Figure 2B). Overall, these results indicate microglial and astrocytic activation, a shift in amyloid precursor protein (APP) processing toward Aβ production, and cell death.

### 2.2. Characterization of Cell Population and EVs Secreted by Amniotic Fluid of Third Trimester

**Figure 3:**
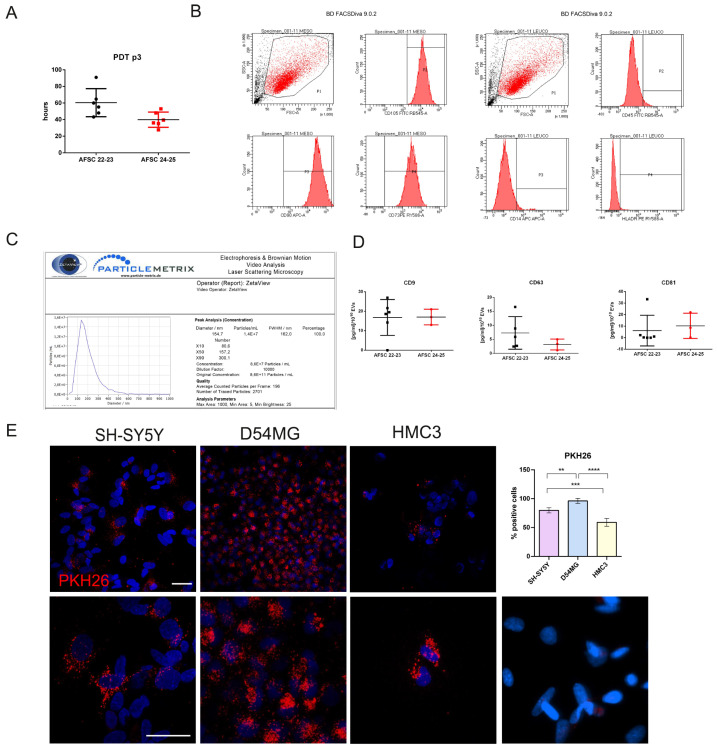
Characterization of human AFSCs and secreted EVs. (**A**) The graph shows the population doubling time (PDT) measured at passage 3 in three cultures of caesarean fluid cells used in this study, compared with five others collected previously (22–23). Values were reported as plot + mean ± SD. (**B**) Representative cytofluorimetric analysis of mesenchymal and leucocytic markers. (**C**) Representative Nanoparticle Tracking Analysis (NTA) analysis performed on EV suspension with ZetaView. (**D**) ELISA analysis of EV markers (CD9, CD81, and CD63) on the three lysates of isolated EVs compared with five others collected previously (22–23). Values were reported as plot + mean ± SD. (**E**) Representative images of immunofluorescence, captured at different magnifications, of SH-SY5Y, D54MG, and HMC3 cells exposed to EVs labeled with PKH26 probe (red). Nuclei are stained blue by DAPI. Scale bars: 10 μm. The last image on the right represents SH-SY5Y cells incubated with the probe alone (without EVs), as negative control. Fluorescence intensity is shown in the graph. Values were reported as mean (*n* = 5) ± SD: ** *p* value < 0.01; *** *p* value < 0.001; **** *p* value < 0.0001.

Amniotic fluid samples were collected from nine Cesarean procedures, performed between the 36th and the 39th week of gestation: the mean maternal age was 36.1 ± 3.6, and the growth rate of these cells, measured as PDT at passage 3, was higher (1.62 ± 0.22 days), but not significantly, than that of cells collected in 2022–2023 (2.69 ± 0.39 days) (Figure 3A).

FACS analysis was performed to evaluate the percentage of positive cells for the three typical mesenchymal markers (CD105, CD90, CD73) in order to define multipotent mesenchymal stromal cells. All the samples of amniotic fluid cells showed more than 90% of positive cells for the three markers (Figure 3B), confirming the high presence of mesenchymal stromal cells, as we previously demonstrated for other samples [18]. We also assessed the expressions of CD45, CD14, and HLA-DR surface molecules, and all markers were negative as expected (<5%).

AFSC-CM samples were processed by concentration and ultracentrifugation to obtain EVs; EVs showed an average size of 152 nm ± 4.6 measured by NTA (like the one of EVs obtained from cells collected in 2022–2023—160 nm), and the EV yield was 5.7 ± 2.1 × 10^9^ particles/10^6^ cells (previously collected—2.1 ± 0.7 × 10^9^ particles/10^6^ cells) (Figure 3C). hAFSC-EVs were positive by ELISA tests for the CD9, CD63, and CD81 tetraspanin signature, as recently published, with levels of 17.0 ± 2.3, 3.2 ± 1.1, and 10.3 ± 6.1 pg/mL/10^10^ EVs, respectively (Figure 3D).

Here, we show that EV exposure in the triple culture resulted in vesicle uptake principally by astrocytes and neurons, but also by microglial cells: a punctate intracellular pattern is typical for PKH26-labeled EV uptake, since EVs are internalized via endocytosis/macropinocytosis and end up in endosomes/lysosomes, which appear as discrete fluorescent puncta (Figure 3E).

### 2.3. Effect of AFSC-EVs on Neurotoxicity Induced by LPS + H_2_O_2_

**Figure 4:**
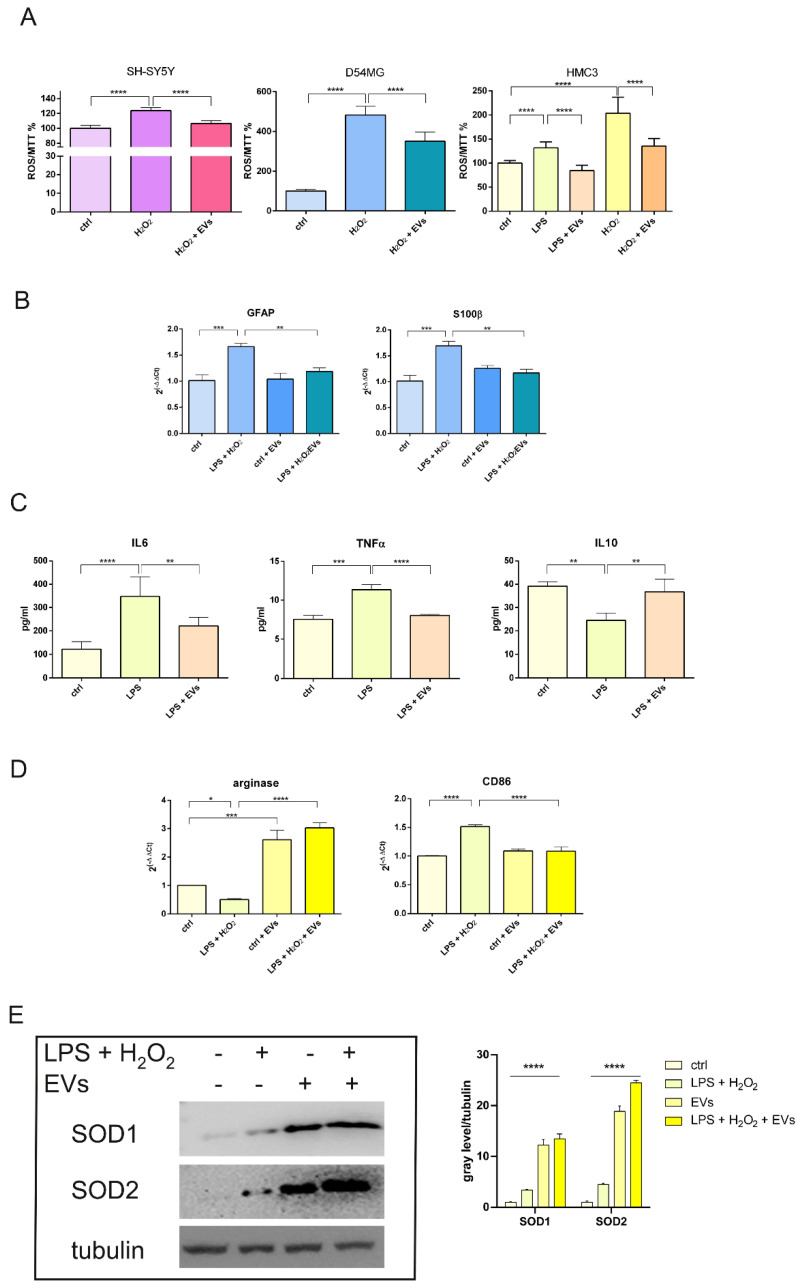
Effect of human AFSC-EV supplementation on neurons, astrocytes, and microglia exposed to LPS and H_2_O_2_ treatments. (**A**) SH-SY5Y, D54MG, and HMC3, pre-treated with AFSC-EVs, were exposed to LPS for 48 h or 250 μM H_2_O_2_ for 3 h (LPS + H_2_O_2_). Graphs represent the intracellular ROS values, evaluated with DCFH-DA probe, normalized to viability values, and analyzed with MTT test. Values were reported as mean (*n* = 3) ± SD: **** *p* value < 0.0001. (**B**) RT-PCR analysis of expression of S100β and GFAP in D54MG cells, pre-treated with AFSC-EVs, after LPS+ H_2_O_2_ treatment. Values were reported as mean (*n* = 3) ± SEM: ** *p* value< 0.01; *** *p* value < 0.001. (**C**) Graphs show the results of ELISA test for IL6, TNFα, and IL10 analyzed in the conditioned medium of HMC3, pre-treated with AFSC-EVs, collected after LPS treatment. Values were reported as mean (*n* = 3) ± SD: ** *p* value < 0.01; *** *p* value < 0.001; **** *p* value < 0.0001. (**D**) RT-PCR analysis of expression of arginase and CD86 in HMC3 cells, pre-treated with AFSC-EVs, after LPS + H_2_O_2_ treatment. Values were reported as mean (*n* = 3) ± SEM: * *p* value < 0.05; *** *p* value < 0.001; **** *p* value < 0.0001. (**E**) Representative images and densitometric analysis of western blot of HMC3, treated or not with EVs, and exposed to LPS + H_2_O_2_, revealed with anti-SOD1, anti-SOD2, and anti-tubulin, as loading control. Values were reported as mean (*n* = 3) ± SD: **** *p* value < 0.0001.

We previously demonstrated that AFSC-EVs exhibit antioxidant properties, as they contain superoxide dismutase (SOD) enzymes [19]. Based on this, we tested the efficacy of EVs against oxidative stress induced by H_2_O_2_: EV treatment significantly reduced ROS levels in neurons, astrocytes, and microglia (Figure 4A). Activation of astrocytes, demonstrated by the enhancement of pan-markers such as GFAP and S100β, was prevented by EVs’ presence (Figure 4B). Interestingly, LPS-induced release of IL6 and of TNFα, and CD86 expression (Figure 4C,D), markers of inflammatory microglial activation, were attenuated by these vesicles. Meanwhile, the expression of arginase and IL10, markers associated with anti-inflammatory microglial phenotype, showed an opposite trend (Figure 4C,D). In addition, the expression of SOD1 and SOD2 was increased in HMC3 cells after EV exposure (Figure 4E), confirming the presence of these scavenging proteins within their cargo. Notably, treatment with LPS + H_2_O_2_ also induced a slight increase in SOD isoform expression in HMC3 cells, suggesting a cellular response to these stressors (Figure 4E).

**Figure 5:**
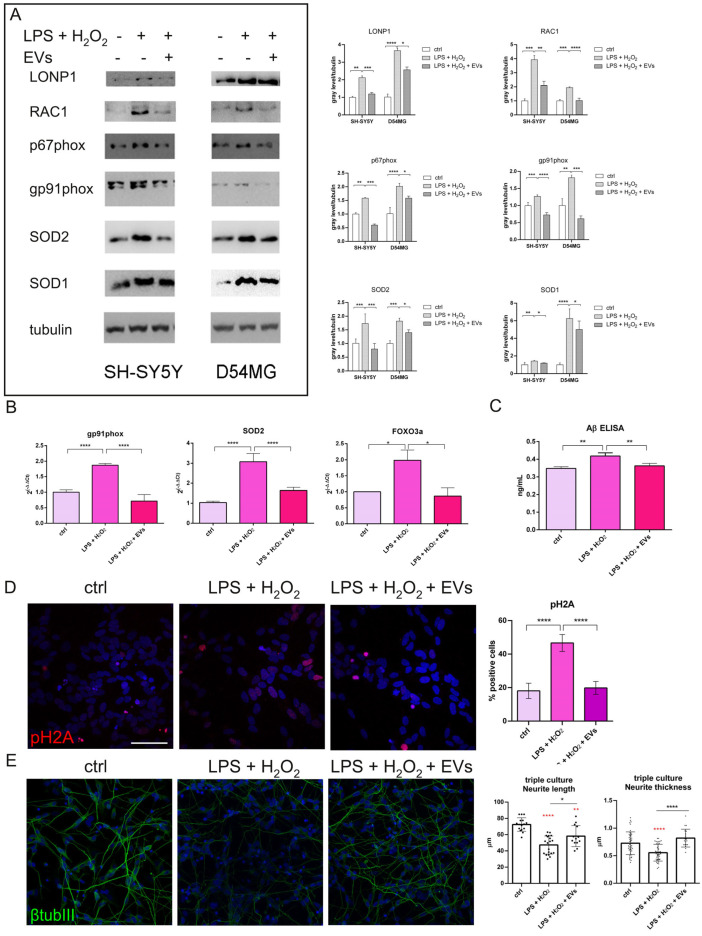
Effect of human AFSC-EV supplementation on the neurotoxicity induced by the combined LPS + H_2_O_2_ treatment. (**A**) Western blot analysis of lysates of SH-SY5Y cells, treated with LPS + H_2_O_2_, with or without the pre-treatment of EVs, revealed with anti-LONP1, anti-RAC1, anti-67phox, anti-gp91phox, anti- anti-SOD1, anti-SOD2, and anti- tubulin, as loading control. Relative densitometric analysis was reported in the graph. Values were reported as mean (*n* = 3) ± SD: * *p* value < 0.05, ** *p* value < 0.01, *** *p* value < 0.001; **** *p* value < 0.0001. (**B**) RT-PCR analysis of expression of FOXO3a, gp91phox, and SOD2 in SH-SY5Y cells, pre-treated with AFSC-EVs, after LPS + H_2_O_2_ treatment. Values were reported as mean (*n* = 3) ± SEM: * *p* value < 0.05; **** *p* value < 0.0001. (**C**) The graph shows the results of ELISA tests for amyloid-β detected in the conditioned medium of the triple culture pre-treated with AFSC-EVs, after LPS + H_2_O_2_ exposure. Values were reported as mean (*n* = 3) ± SD: ** *p* value < 0.01. (**D**) The graph represents the analysis of immunofluorescence images of SH-SY5Y cells, treated or not with LPS + H_2_O_2_, with or without EVs, stained with DAPI (blue) and anti-pH2A (red). Scale bar: 10 μm. Values were reported as mean (*n* = 3) ± SD: **** *p* value < 0.0001. (**E**) Immunofluorescence images of SH-SY5Y cells, treated or not with LPS + H_2_O_2_ with or without EVs, stained with DAPI (blue) and anti-βtubulin III (green). The graphs represent the length and the thickness of neurites. Scale bar: 10 μm. Values were reported as mean (*n* = 3) ± SD: * *p* value < 0.05, ** *p* value < 0.01, *** *p* value < 0.001, **** *p* value < 0.0001.

NOX2 (NADPH oxidase 2) is one of the major enzymatic sources of reactive oxygen species (ROS) in the brain, generating superoxide anion. Increased NOX2 activity has been reported in both Alzheimer’s disease and cerebrovascular disorders, where it contributes to amyloid toxicity, endothelial dysfunction, and chronic neuroinflammation [20]. In triple-culture, both in neurons and astrocytes, WB analysis indicated that the ROS production induced by LPS + H_2_O_2_ may originate from NOX 2, as its subunits gp91phox, p67phox, and RAC1 were upregulated (Figure 5A). This activation led to cellular defense responses, including SOD isoforms’ increase, particularly the mitochondrial form: both SOD2 and LONP1, a mitochondrial protease for oxidized proteins, were enhanced (Figure 5A). Moreover, RT-PCR analysis confirmed SOD2 modulation downstream of FOXO3a (Figure 5B). FOXO3a is activated under oxidative or metabolic stress through dephosphorylation and nuclear translocation, where it can induce the expression of protective genes such as catalase and SOD2. The rationale for examining NOX2 and FOXO3a in mixed dementia is that these markers capture two interconnected processes central to disease pathogenesis: NOX2 as a driver of neurovascular oxidative injury and inflammation, and FOXO3a as a master regulator of stress adaptation, antioxidant defense, and cell fate. In neurodegenerative settings, FOXO3a has been implicated in neuronal resilience, aging, and microglial responses, making it particularly relevant in dementia models where oxidative and inflammatory stress are prominent [21]. Their modulation can therefore help clarify the molecular mechanisms underlying disease progression and therapeutic responses. Interestingly, ELISA tests demonstrated that amyloid-β accumulation observed in the conditioned medium under LPS + H_2_O_2_ treatment was counteracted in EV-treated samples (Figure 5C), consistent with decreases in DNA damage, as measured by pH2A staining (Figure 5D) and improvements in neurite morphology (Figure 5E). Finally, we observed a reduction in the neuronal cell death in the presence of EVs: the mean cell death values (in %) were 10.25 ± 1.33 for controls, 20.50 ± 2.51 for LPS + H_2_O_2_-treated samples, and 15.10 ± 1.66 for EV-treated samples (different from the LPS + H_2_O_2_-treated samples: ** *p* value < 0.01).

### 2.4. Modulation of Neuronal Mitochondria

**Figure 6:**
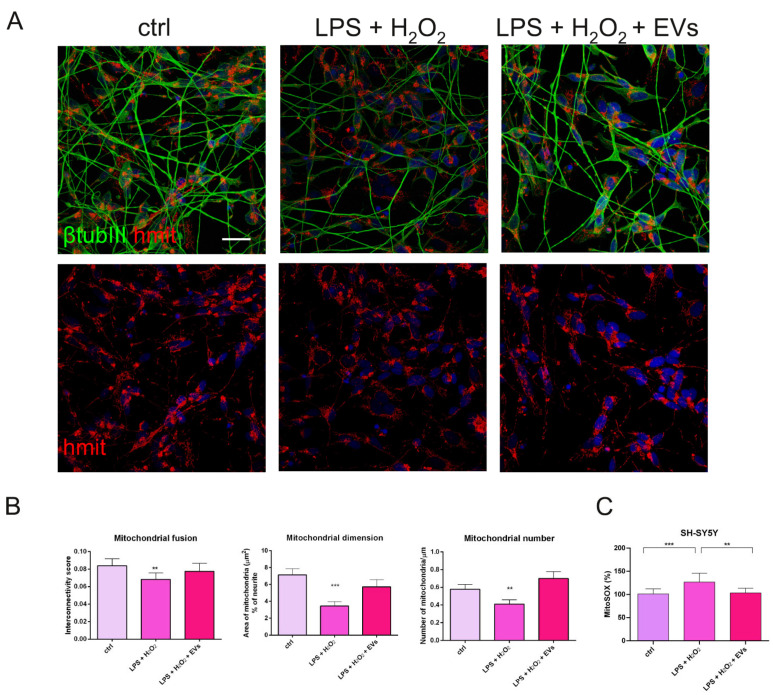
Effect of human AFSC-EV supplementation on the mitochondrial defects of neurons induced by the combined LPS + H_2_O_2_ treatment. (**A**) Representative immunofluorescence images of neuron cells, incubated or not with EVs, and in culture with astrocytes and microglia, all exposed to LPS and H_2_O_2_, were stained with DAPI (blue), βtubIII (green), and h-mit (red). Superimposed images are shown on the top. Scale bar: 10 µm. (**B**) Graphs showing the mitochondrial skeleton analysis on the area, the number, and the interconnectivity index. Values were reported as mean (*n* = 3) ± SD: ** *p* value < 0.01; *** *p* value < 0.001. (**C**) Graph represents the mitochondrial superoxide values, evaluated with MitoSOX probe. Values were reported as mean (*n* = 3) ± SD: ** *p* value < 0.01; *** *p* value < 0.001.

In this system, mitochondrial dynamics, including fission and fusion processes, play a crucial role in maintaining neuronal homeostasis and survival. Excessive fission or impaired fusion can lead to mitochondrial fragmentation, loss of membrane potential, and increased production of ROS, ultimately resulting in neuronal damage [22]. Immunofluorescence analysis showed that mitochondrial organization was affected following LPS + H_2_O_2_ exposure (Figure 6A) in our model of mixed dementia. This treatment led to a decrease in mitochondrial fusion, area, and number, as demonstrated by mitochondrial morphological analysis (Figure 6B). The mitochondria-targeted derivative MitoSOX allows detection of mitochondrial superoxide. The increase observed after LPS + H_2_O_2_ exposure was rescued by EV pretreatment (Figure 6C). Consistently, the presence of EVs appeared to mitigate these effects by preserving mitochondrial function, limiting mitochondrial oxidative stress, and protecting neurons from structural damage.

## 3. Discussion

The brain is prone to oxidative stress-induced cell damage due to its high oxygen demand, the abundance of redox-active metals, and relatively high levels of oxidizable polyunsaturated fatty acids. Excessive oxidative stress leads to neuronal cell death, a key physiological process in several neurological disorders, including neurodegenerative diseases, stroke, and traumatic brain injury [23]. Such redox dysregulation is a common pathophysiologic event downstream of inflammation and hypoxia, contributing to the onset of age-related brain diseases. A growing body of evidence suggests that several systemic factors act together to produce AD [24]. Accordingly, β-amyloid (Aβ) and abnormal tau are unlikely to represent the primary cause of sporadic AD, but rather, both may arise downstream of unrelated primary pathological processes [25], including neuroinflammation and hypoxia. Neuroinflammation is typically activated by the bacterial endotoxin lipopolysaccharide (LPS). However, since LPS does not enter the brain under physiological conditions when administered alone [26], additional factors are likely required to facilitate LPS entry into the aging brain. These include ischemia, hypoxia, and other factors that cause blood–brain barrier (BBB) disruption. Areas of ischemic and/or hypoxic injury might provide a portal for LPS entry from the bloodstream into the human brain since, in genetic mouse models of AD, alterations of blood flow and BBB permeability occur prior to both symptoms and Aβ deposition. Moreover, AD has been associated with several different infections, which might act as potential triggers [27]. Notably, cerebral vascular disease and AD pathology co-exist in up to 80% of aging human brains [28]. Overlapping features of AD and vascular dementia (VaD) characterize mixed dementia (MD), representing the most prevalent form of late-life cognitive decline.

To mimic MD in vitro, we combined LPS and H_2_O_2_ treatments to recapitulate key pathological features of AD and ischemic injury, respectively, including Aβ deposition and oxidative stress. As the crucial mediator of oxidative stress, hydrogen peroxide and its sources lead to lipid peroxidation, DNA damage, and mitochondrial dysfunction, causing neuronal cell death [4]. We therefore hypothesized that neuronal and glial responses to these stressors could involve mitochondrial antioxidant machinery. Stress-induced regulation of SOD2 transcript and protein levels was examined in differentiated cholinergic neurons produced from human SH-SY5Y neuroblastoma cells (maturation induced by 7-day supplementation with retinoic acid) [29], human D54MG astrocytoma cells, as a model for brain astroglia, and the human microglial cell line HMC3, to mimic the neural macrophagic cell populations. Although this model does not reach the complexity of the human brain, lacking the vascular cell component and oligodendrocytes, it allows the simultaneous evaluation of BBB disruption-related effects, namely oxidative stress and LPS entry, on the principal cell actors involved in neurodegeneration. Neurons were exposed to LPS-induced neuroinflammation, as LPS is a potent inflammatory stimulus that activates microglia (HMC3). LPS directly triggers pro-inflammatory gene expression in astrocytes as well: for example, in rat cortical astrocytes, LPS markedly increased mRNA levels of inflammatory mediators such as IL1β and IL6 [30]. Moreover, LPS treatment has been reported to dramatically lower the viability and decrease BDNF levels in SH-SY5Y cells while elevating the pro-inflammatory cytokines IL6 and TNF-α [6]. Furthermore, Aβ deposition and cytotoxic effects have been observed in SH-SY5Y cells cultured alone and treated with LPS (from 0.5 to 10 µg/mL) [31]. Collectively, LPS was used in this model to induce Aβ expression and aggregation, inflammatory processes, and neurotoxicity by its combined action on neurons, astrocytes, and microglial cells.

On the other hand, high levels of ROS, like H_2_O_2_, are associated with brain ischemic injury and the induction of toxic effects such as DNA damage and neurite degeneration in neuronal cells. BBB-forming cells, like endothelial cells and astrocytes, are among the first exposed to hypoxia and reperfusion events. In response, astrocytes become reactive and release excessive H_2_O_2_ and nitric oxide [32]. Here, LPS-exposed astrocytes and microglia cells were treated with H_2_O_2_, thus amplifying neuroinflammation, AD-related protein aggregation, and creating a “feed-forward loop” where neuronal and vascular damage reinforce each other.

Therefore, the first goal of this study was to validate the in vitro model of mixed dementia, showing how each cell type, alone or in co-culture, responds to LPS, H_2_O_2_, or both exposures.

LPS 1 µg/mL was incubated for 48 h, as we previously reported that several inflammatory events may initiate the AD onset. As demonstrated by ELISA tests, cytofluorimetric analysis, and immunofluorescence imaging, LPS alone induced Aβ accumulation, affected neurite morphology, and caused neuronal cell death. An acute 250 µM H_2_O_2_ exposure for 3 h, chosen to mimic an ischemic event, caused neurite morphological defects and neuronal death as well, but through DNA damage. Interestingly, the combination of these stressors did not produce a synergistic effect in either mono-culture or co-culture conditions. We can speculate that the pre-treatment with LPS may have a priming effect on neurons, preparing them for the second stressful event. This biological phenomenon of inflammatory preconditioning (or hormesis) may be induced by the exposure to LPS acting as a sub-lethal priming stimulus that triggers the cell’s endogenous defense mechanisms. By the time the H_2_O_2_ is administered, the cells may have already upregulated their antioxidant and cytoprotective pathways, making them more resilient to subsequent oxidative stress. This could explain why SH-SY5Y cell death does not increase in the presence of both treatments compared to the H_2_O_2_ alone.

The co-culture condition shows that LPS treatment worsened neuronal viability by activating microglial cells to secrete other RONS and cytokines, namely IL6 and TNFα. In contrast, H_2_O_2_ treatment was less detrimental to neurons in the presence of different cytotypes, possibly due to a ROS-scavenging action exerted by astrocytes and microglial cells. Based on these considerations, this may explain the reduced harmful effect observed on SH-SY5Y in co-culture when exposed to both stressors.

We observed a dramatic upregulation of SOD1, and even more prominently, of SOD2 in neurons and astrocytes as a result of a defensive response of these cells to the stressors. Moreover, RT-PCR analysis revealed that the FOXO3a pathway was activated in the presence of MD stimuli. However, looking at the single exposure to LPS or H_2_O_2_, it is interesting to note that LPS alone was the main inducer of SOD upregulation, whereas ROS levels in SH-SY5Y remained unchanged, and H_2_O_2_ exposure (3 h only) did not induce this modification in SOD expression (Supplementary Figure S1). This observation prompted us to investigate the mechanism behind LPS-induced antioxidant enzyme upregulation. It has been found that NADPH oxidase 2 (NOX2, gp91phox), a typical source of ROS constitutively expressed in neurons, is significantly increased in the brain of ageing mice and is associated with vascular and inflammatory processes, including AD development and progression [33]. In our study, NOX2 subunits were upregulated in LPS-treated neurons, consistent with the prior study by Sun, where it was demonstrated that LPS-induced neuronal damage similarly progresses through NOX2-mediated ROS production [34]. Beyond neurons, NOX2 was highly expressed in microglia and astrocytes, supporting its central role in the oxidative stress generation within our triple-culture system. Moreover, NOX2 was demonstrated to be involved in vascular complications underlying AD, specifically those affecting cerebral blood flow, and in inflammatory crosstalk between microglial and neuronal cells. In this context, microglial activation induces the expression and activation of neuronal NOX2, in addition to mitochondrial dysfunction.

Oxidative stress observed in depression suggests that an excess of superoxide anion radicals (O_2_^−^), generated in mitochondria or by NOX2, may play crucial roles in mediating mitochondrial damage, inducing an intriguing form of mitochondrial damage, namely mitochondrial peripheral fission [35]. Dysregulation of mitochondrial dynamics, particularly excessive fission, leads to mitochondrial fragmentation and impairments in its function, contributing to the occurrence of neurodegenerative diseases, stroke, and VCI [36]. Mitochondrial morphology and function have been investigated in the present study, revealing that LPS + H_2_O_2_ treatment caused a less fused profile and a reduction in mitochondrial number, suggesting a link between neuronal ROS generation and mitochondrial dysfunction. Interestingly, Mito-apocynin, a mitochondria-targeted inhibitor of NOX2 activity, has been demonstrated to restore mitochondrial morphology and function in damaged mitochondria of neuronal N27 cells [37], supporting the role of NOX2-derived ROS in neuronal mitochondria dysfunction. Moreover, the literature confirms that β-amyloid, the most active among the misfolded proteins, activates ROS production in microglia and astrocytes via the activation of NADPH oxidase [38]. Here, we further show that this activation also occurs in neurons and may induce mitochondrial dysfunction, neurite defects, DNA damage, and neuronal cell death.

Extracellular vesicles (EVs) are increasingly considered a promising cell-free alternative to direct stem cell transplantation, as they are generally less immunogenic and easier to standardize than viable stem cell products, making them attractive candidates for translational medicine [39]. Standardization of manufacturing, storage, dosing, and quality control remains essential before widespread therapeutic implementation. In our experimental condition, EV production may be influenced by the physiological state of the parental cells, including donor variability. All donors were healthy, and amniotic fluids were collected through caesarean section between the 38th and 39th week of gestation, and the procedures used to obtain conditioned medium (CM) and subsequently isolate EVs were identical for all samples.

Thus, we investigated how a single yet complex cell-derived therapeutic tool can exert beneficial effects on these events involved in mitochondrial dysfunctions and cell death across different brain cell types. Indeed, EVs secreted by mesenchymal stem cells have a composite cargo difficult to deeply characterize; however, they could reach the brain via systemic circulation both before and after BBB disruption. Once in the brain, EVs can fuse with all the cytotypes or exert their effects through the modulation of specific target cells. Here, we demonstrate that, in our model, AFSC-EVs were taken up by neurons, astrocytes, and, to a lesser extent, also by microglial cells. This difference may be explained by the different times of the EV-dye incubation among the cell types: indeed, we performed the analysis following the experimental scheme. Thus, the EV-dye was incubated for 48 h with SH-SY5Y and D54MG, while only for 24 h with HMC3.

In this study, we do not provide experimental evidence identifying the specific molecules contained within the EVs that are responsible for protecting neurons from the neurotoxic effects induced in our experimental system. However, based on our previous studies on these EVs and on the existing literature, we propose the following speculative considerations.

We previously demonstrated that AFSC-EVs exhibit antioxidant properties in terms of proteins and miRNAs [18]. Therefore, they could counteract the oxidative stress caused by accumulated H_2_O_2_ as a neuropathogenic factor. In addition, they can also modulate neuroinflammation, as their anti-inflammatory potential has been demonstrated in several systems [12,40]. This approach adheres to our proposal that handling ROS, either directly or by boosting protective cellular mechanisms, could be invaluable as a novel AD and MD therapy.

Comparing the NOX2-ROS literature with our previously published data on the most abundant miRNA found in amniotic fluid cells obtained from term pregnancies [18], we identified miR-181b as a highly expressed miRNA reported to affect NOX2 activity. Indeed, miR-181b has been postulated to elicit its effects via NOX2 suppression. However, a direct miR181b–NOX2 mRNA interaction has not been demonstrated [41]. Other NOX2 targeted miRNAs, namely miR-652 and miR-532 [42], were also present, but not with high abundance. The influence of other EV-associated miRNAs on oxidative stress, such as miR-320a and miR-214, can have important functions in decreasing the production of sirtuin 4 and suppressing the expression of calcium/calmodulin-dependent protein kinase II, respectively [43,44]. In our study, these miRNAs were detected in EVs, although they were not among the top 10 most abundant. These mechanisms could lead to a reduction in the formation of ROS. Similarly, miR-495, known to upregulate SOD2 expression and to promote the intracellular survival of BCG/H37Rv through a decline in ROS levels [45], may play a role as well.

Regarding the modulation of microglia, two miRNAs highly expressed in caesarean AFSC-EVs, namely miR-21-5p and miR-181b (in the top 10, the first and the ninth) [18], have been demonstrated to alleviate neuroinflammation by inhibiting M1 polarization of microglia [46,47]. Therefore, we propose that AFSC-EVs, thanks to their miRNA cargo, can protect neurons also by repressing M1 inflammatory microglial activation and promoting an M2 microglia phenotype, as we observed by the changes of CD86, IL10, and arginase expression, respectively, in EV-treated HMC3.

However, AFSC-EVs were found to contain other highly expressed miRNAs that can promote NOX2 activity, such as miR-125b and 34a [48], which can potentially mediate redox imbalance and induce mitochondrial dysfunction. Surprisingly, EV levels of miR-34a and miR-125b-5p have been reported to decrease in AD patients [49,50]. All these considerations do not confer the miRNA content of AFSC-vesicles a decisive role in modulating the redox state. Rather, the already demonstrated protein content [18,51], characterized by enzymes such as SOD2, SOD1, and catalase, is more convincing. Moreover, MSC-EVs protein content is known to possess an anti-inflammatory potential, as we previously demonstrated for AFSC-EVs as well (TGFβ, IDO, and HGF) [19]. Indeed, MSC-EVs contain many neurotrophic factors (NTFs), immunomodulatory, and anti-inflammatory molecules, including transforming growth factor-β (TGFβ), that promote functional recovery [52], reducing neuroinflammation and improving cognitive impairment in different animal models of neurological diseases [53].

It was not surprising that, besides SOD2, another protein was upregulated after LPS/H_2_O_2_ treatment; LONP1, a nuclear-encoded mitochondrial chaperone protein, involved in generating the mitochondrial unfolded protein response (mtUPR) [54]. Cells respond to the accumulation of unfolded, misfolded, or damaged proteins by conducting protein quality control (PQC) and upregulating the expression of LONP1 to degrade defective proteins. Notably, AFSC-EVs prevented neuronal mitochondria damage by increasing their fusion and protecting their morphological structure and function. Although elevation of SOD isoforms in neurons and astrocytes was not entirely prevented by the treatment with EVs, this is consistent with the demonstration that EVs themselves carry these antioxidant proteins, as experiments on EV-treated microglial cells demonstrated. In fact, we cannot distinguish between SOD proteins derived from EVs and those produced endogenously by recipient cells. Notably, we observed a relevant decrease in neuronal SOD2 and FOXO3a mRNA levels, indicating that the cells may not require further activation of the antioxidant machinery. In contrast, the expression of NOX2 subunits was deeply modulated by AFSC-EVs treatment, suggesting that processes upstream NOX2 activation were inhibited by EVs, thereby avoiding oxidative stress generation. Consistently, increases in ROS concentration in neurons and astrocytes, as well as IL6 and TNFα production from microglia, were prevented. On the other hand, microglia exposed to EVs highly expressed anti-inflammatory markers, such as arginase and IL10. Interestingly, this protective effect against oxidative damage also extended to neurite morphology, Aβ accumulation, and cell viability, confirming that EVs exerted cytoprotective and neurite-preserving effects. Specific experiments should be performed to prove that EV-derived SOD proteins and/or miRNAs are responsible for the observed neuroprotective effects. Moreover, functional readouts such as synaptic markers or electrophysiological activity would further strengthen the evidence supporting neuroprotection. Additional considerations should be taken into account when interpreting ROS measurements. DCFH-DA has limitations in specificity and does not directly react with H_2_O_2_. Moreover, the intermediate DCF radical can react with molecular oxygen, generating superoxide and H_2_O_2_, meaning the probe may artificially amplify oxidative signals. Nevertheless, it remains widely used as a general indicator of intracellular oxidative stress and redox-signaling changes. Similarly, DHE and its mitochondria-targeted derivative MitoSOX are commonly used as superoxide probes but can undergo non-specific oxidation by many oxidant species [55]. Therefore, these probes should be interpreted as indicators of intracellular oxidative changes rather than precise quantitative measurements of ROS species. To minimize these limitations, we employed multiple ROS probes with different chemistries or different localization/specificity (DCFH-DA, DHE, and MitoSOX), to verify that our results were consistent with endogenous production of ROS and cell oxidation. Furthermore, the present study was designed as an initial mechanistic evaluation in a multicellular in vitro model based on SH-SY5Y cells, which have limited neuronal maturity compared with primary neurons or iPSC-derived neurons.

## 4. Materials and Methods

### 4.1. Amniotic Fluid Stem Cell Isolation and Culture

Human AFSCs were obtained from nine amniotic fluid samples from healthy white human donors, collected during full-term C-sections at the Unit of Obstetrics & Gynecology, at the Policlinico Hospital of Modena (Italy), all with patient consent as well as institutional ethical approval (protocol 360/2017 dated 15 December 2017, approved by Area Vasta Emilia Nord). In general, the time between collection and processing was kept as short as possible to minimize cell death. First, cells were collected by gradient Ficoll separation, then washed with PBS and centrifuged at 300× *g* for 5 min [18].

The supernatant was discarded, and the pellet was washed again with PBS and dissolved in Ammonium chloride to reach 0.8% to lyse the remaining erythrocytes. Thereafter, the cell solution was incubated at 4 °C for 20 min and centrifuged again. This procedure was repeated until the pellet had a clear color. Afterward, the cells were cultured in culture medium (αMEM), supplemented with 20% (*v*/*v*) fetal bovine serum (FBS), 2 mM of L-glutamine, 100 U/mL penicillin, and 100 μg/mL streptomycin (all from EuroClone Spa, Milano, Italy). Once attached, the colonies were visible after 7–10 days, and the medium was changed.

Cells were seeded in a p60 petri dish at a density of 5000 cells/cm^2^, cultured for 7 days, then detached, counted, and seeded again at 5000 cells/cm^2^. Cultures were performed until passage 8, and population doubling time (PDT) for each passage was measured by applying the formula reported in [56].

### 4.2. FACS Analyses

Adherent cells were harvested for surface antigen analysis. Briefly, cells were detached from plastic support by trypsin (EuroClone Spa, Milano, Italy), counted, and aliquoted in FACS analysis polypropylene tubes (0.5–1 × 10^6^ cells/tube) (VWR, Milan, Italy). Cells were subsequently incubated in blocking buffer (100 μL each 0.5–1 × 10^6^ cells) containing Dulbecco’s Modified Eagle’s Medium (DMEM, Gibco from Sigma–Aldrich, St. Louis, MO, USA), 10% FBS (PAA Laboratories now GE Healthcare, Irvine, CA, USA), and 0.1 M of sodium azide and human immunoglobulin G (both from Sigma) and incubated for 20′ on ice. After a PBS (PAA Laboratories) washing step, cells were resuspended in PBS (PAA Laboratories) with 0.5% bovine serum albumin (BSA, Sigma–Aldrich, St. Louis, MO, USA) and stained on ice and in the dark for 30′ with the following monoclonal antibodies: APC-anti-CD45, FITC-anti-HLADR, PE-anti-CD14, (all from Becton Dickinson, Franklin Lakes, NJ, USA); APC-anti-CD90, FITC-anti-CD105, PE-anti-CD73 (all from BD Pharmingen, Franklin Lakes, NJ, USA). In all the experiments, the corresponding isotype-matched antibodies were used as negative controls (BD Pharmingen and Becton Dickinson, Franklin Lakes, NJ, USA). Data were collected using a FACS Aria III flow cytometer (BD Biosciences, Franklin Lakes, NJ, USA) and analyzed on FACS Diva software (Version 9.0, BD Biosciences, Franklin Lakes, NJ, USA).

### 4.3. Extracellular Vesicles Isolation

Human AFSCs grew in 150 cm^2^ flasks until sub-confluence (around 2 × 10^6^ cells). Then, cells were maintained in FBS-free culture medium (18 mL) for 4 days to avoid contamination with EVs derived from the FBS solution. The collected conditioned medium (CM) was centrifuged at 300× *g* for 10 min at 4 °C to eliminate cellular debris and then concentrated up to 6 mL by using centrifugal filter units with a 3K cut-off (Merck Millipore, Burlington, MA, USA). The concentrated CM was again centrifuged at 10,000× *g* for 30 min at 4 °C, and then, the supernatant was ultracentrifuged in polypropylene ultracentrifuge tubes (13.5 mL, Beckman Coulter) at 100,000× *g* for 90 min at 4 °C in a Beckman Coulter Optima L-90 K centrifuge (SW-41 rotor); the supernatants were discarded and the pellets were resuspended in 13 mL DPBS (Corning, Manassas, VA, USA) and ultracentrifuged again (100,000× *g*, 90 min at 4 °C) [18]. The pellets, resuspended in a ratio of 18 mL to 25 µL of DPBS, were stored at −80 °C. After dilution 1:1000, the size distribution and concentration of EVs were analyzed by nanoparticle tracking analysis using a ZetaView particle tracker from ParticleMetrix (Ammersee, Germany).

### 4.4. Cell Line Culture

The SH-SY5Y human neuron cell line (kindly provided by Prof. Corsi, Unimore) was grown in high-glucose DMEM supplemented with 10% (*v*/*v*) of FBS, 2 mM of L-glutamine, 100 U/mL of penicillin, and 100 µg/mL of streptomycin (all from EuroClone Spa, Milano, Italy). Neuronal differentiation was induced in high-glucose DMEM supplemented with 0.5% of FBS and 10 μM of all-trans retinoic acid for 7 days.

HMC3 human microglial cells were purchased from ATCC (#ATCC-CRL-3304, Manassas, VA, USA). Cells were cultured in DMEM-F12 supplemented with 10% (*v*/*v*) of low-endotoxin FBS (Euroclone, Milano, Italy), 2 mM of L-glutamine, 100 U/mL of penicillin, and 100 μg/mL of streptomycin. The cells were pre-activated with 1 μg/mL LPS (Sigma Aldrich, St. Louis, MO, USA) for 24 h prior to infection.

D54MG human astrocyte cell line (kindly provided by Prof. Cermelli, Unimore) was grown in DMEM-F12 supplemented with 10% (*v*/*v*) of FBS, 2 mM of L-glutamine, 100 U/mL of penicillin, and 100 μg/mL of streptomycin. All cells were maintained in a humidified incubator at 37 °C with 5% CO_2_.

### 4.5. Triple Culture Experiments

We developed a three-culture system exploiting the use of well-known transwell supports (Cell culture insert 1.0 µm pore size, 6-well, Falcon, Corning, NY, USA). We used an experimental co-culture model designed to maintain the different cell populations in separate but communicating compartments.

**Figure 7:**
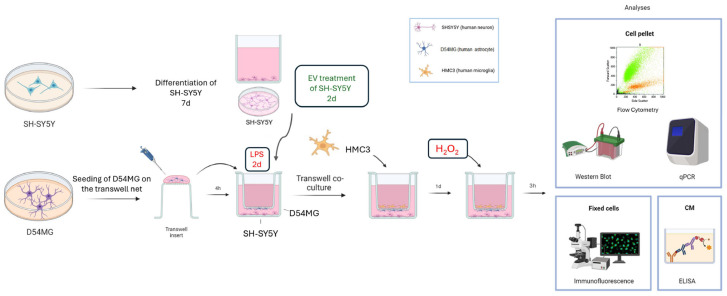
Scheme of experimental design. Transwell-based triple-culture system comprising SH-SY5Y, D54MG and HMC3. Cells were treated with LPS (48 h for SH-SY5Y and D54MG, 24 h for HMC3), and H_2_O_2_ (3 h), followed by subsequent analyses.

Human neuronal cells (SH-SY5Y, 2.5 × 10^5^) were seeded on the bottom of the well, allowing them to grow individually, and then differentiated for 1 week in differentiation medium before the introduction of the other cell types. Upon differentiation with retinoic acid (RA), neuronal cells undergo growth arrest, extend neurites, and acquire a more mature neuronal-like phenotype, thereby reducing features associated with the undifferentiated malignant state. Meanwhile, astrocytes (D54MG) were seeded on the insert positioned upside down, where they rapidly adhered to the substrate. Once attached, the insert containing astrocytes was placed into the well containing the differentiated SH-SY5Y cells. After 24 h, microglial cells (HMC3) were seeded on the apical side of the transwell insert. The cell number ratio was 4:2:1 (SH-SY5Y: D54MG: HMC3). This configuration enabled indirect interaction among neuronal-like cells, astrocytes, and microglia through soluble factors while keeping each population physically separated and allowing better control of cell-specific responses. Cells, in a total volume of 3 mL, were exposed to 1 µg/mL LPS after the treatment with pooled samples of extracellular vesicles (4 × 10^9^ particles), previously isolated by ultracentrifugation from the conditioned medium culture of human AFSCs. Forty-eight hours later, cells underwent acute H_2_O_2_ treatment, followed by analysis on the triple-culture (see Figure 7).

### 4.6. EVs Labelling

For uptake studies, purified EVs were labeled with a PKH26 (Sigma-Aldrich, St. Louis, MO, USA) according to the manufacturer’s protocol. Briefly, the EV suspension was incubated with 4 µL of 10 µM PKH26 dye (λex 551 nm; λem 567 nm) for 5 min at room temperature. Then, the suspension was filtered through a 100 kDa filter by centrifuging at 10,000× *g* for 5 min to remove the excess dye. Labeled EVs were collected by centrifuging again the filter upside down, then the EVs were resuspended in serum-free medium and co-cultured for 24 h with HMC3 and for 48 h with SH-SY5Y and D54MG, as reported in the experimental scheme. Cells were fixed, then exposed to DAPI staining and visualized with laser scanning confocal microscopy, Nikon A1. (Nikon Corporation, Tokyo, Japan) Cells incubated with the dye alone have been considered as negative control.

### 4.7. MTT Assay

Cells (D54MG, HMC3 and differentiated SH-SY5Y) were seeded in 96-well plates at the density of 10^4^ cells/well in 100 μL of culture medium, with four replicates for each condition. At different time points after treatment, 0.5 mg/mL MTT was added and incubated for 3 h at 37 °C. After incubation, the medium was removed, and acidified isopropanol was added to solubilize the formazan salts. The absorbance was measured at 570 nm using a microplate spectrophotometer (Appliskan, Thermo Fisher Scientific, Vantaa, Finland).

### 4.8. ROS Detection

To evaluate intracellular ROS levels, dichlorodihydrofluorescein diacetate (DCFH-DA) assay was performed similarly to what was previously described [57]. Cells (D54MG, HMC3, and SH-SY5Y) were seeded in a 96-well black/clear bottom plate with five replicates for each condition, at a density of 10^4^ cells/well. After cell treatments, cell culture medium was removed, and 5 μM of DCFH-DA was incubated in PBS for 30 min, at 37 °C and 5% CO_2_. The cell culture plate was washed with PBS, and cell fluorescence was read at 485 nm (excitation) and 535 nm (emission) using the multiwall reader Appliskan (Thermo Fisher Scientific, Vantaa, Finland).

Similarly, to evaluate intracellular superoxide, Dihydroethidium (Hydroethidine) probe (Invitrogen, Waltham, MA, USA) was used. After treatment, cells were washed two times with PBS and incubated for 30 min at 37 °C and 5% CO_2_ with 10 µM of DHE diluted in PBS. After washing, cell fluorescence was read at 302/518 nm (excitation) and 605 nm (emission) using the multiwall reader Appliskan (Thermo Fisher Scientific, Vantaa, Finland).

Mitochondrial superoxide was evaluated by MitoSOX^TM^ (Invitrogen, Waltham, MA, USA). After treatment, cells were washed and then incubated with 5 μM of MitoSOX diluted in PBS for 10 min at 37 °C and 5% CO_2_. Cells were gently washed, and cell fluorescence was read at 510 (excitation) and 580 (emission) using the multiwall reader Appliskan (Thermo Fisher Scientific, Vantaa, Finland).

Cellular autofluorescence was subtracted as a background using the values of the wells not incubated with the probe.

### 4.9. Immunofluorescence and Confocal Microscopy

For immunofluorescence analysis, cells seeded on coverslips or on the transwell membrane were processed, and confocal imaging was performed using a Nikon A1 confocal laser scanning microscope, as previously described [58].

Primary antibodies to detect β-TubulinIII, pH2A, and human-mitochondria (Merck Millipore, MA, USA), as well as Rab5 (Santa Cruz Biotechnology, Biotechnology, CA, USA) and βamyloid (6E10, BioLegend, London, UK), were used following datasheet-recommended dilutions. Alexa secondary antibodies (Thermo Fisher Scientific, Waltham, MA, USA) were used at a 1:200 dilution.

The confocal serial sections were processed with ImageJ-1.51 software to obtain three-dimensional projections. The image rendering was performed by Adobe Photoshop software (CS&Portable).

### 4.10. Mitochondria Morphological Analysis

Mitochondrial analyses were performed using ImageJ software (ImageJ 1.54g). Briefly, confocal images labelled with h-mitochondria (hmit) (Merck Millipore, Billerica, MA, USA) were pre-processed using unsharp mask and median filter to enhance image quality. Then, particles were analyzed on binary images based on their number, dimension, and perimeter. Subsequently, the interconnectivity was calculated as previously described by Wiemerslage and Lee [59].

### 4.11. RNA Isolation and Quantification

RNA was isolated using TRIzol™ Reagent (Invitrogen, Waltham, MA, USA) following the manufacturer’s protocol. Starting from 1 μg of the extracted RNA, the cDNA was obtained using SensiFAST™ cDNA Synthesis Kit (Meridian Life Science Inc., Cincinnati, OH, USA) following the manufacturer’s protocol. Real-time PCR was performed using SensiFAST SYBR Hi-ROX Kit following the manufacturer’s protocol (Meridian Life Science Inc., Cincinnati, OH, USA). Real-time PCR reaction was carried out in a total volume of 20 μL, loading 250 ng of cDNA and 500 nM of each primer. cDNA amplification was performed by activating the polymerase for 30 s at 95 °C, followed by 40 cycles of 5 s at 95 °C and 30 s at 60 °C. Normalized expression levels were calculated relative to control cells according to the ΔCT method. Primer sequences used in this study are listed below:
**Gene****Forward****Reverse***Actin*CACCATTGGCAATGAGCGGTTCAGGTCTTTGCGGATGTCCACGT*GAPDH*GTCTCCTCTGACTTCAACAGCGACCACCCTGTTGCTGTAGCCAA*CD86*CCATCAGCTTGTCTGTTTCATTCCGCTGTAATCCAAGGAATGTGGTC*FOXO3a*TCTACGAGTGGATGGTGCGTTGCTCTTGCCAGTTCCCTCATTCTG*Arginase1*TCATCTGGGTGGATGCTCACACGAGAATCCTGGCACATCGGGAA*GFAP*CTGGAGAGGAAGATTGAGTCGCACGTCAAGCTCCACATGGACCT*Gp91-phox*GTCACACCCTTCGCATCCATTCTCAAGTCAGTCTGAGACTCATCCCAGCCAGTGAGGTAG*S100β*GAAGAAATCCGAACTGAAGGAGCTCCTGGAAGTCACATTCGCCGT

### 4.12. ELISA Assays

IL6, IL10, and TNFα (from Cusabio Technology, Houston, TX, USA) were quantified in the conditioned medium collected from transwell co-culture by using ELISA assays, according to the manufacturer’s instructions. Samples were run in triplicate. A standard curve was constructed using known concentrations of recombinant human and murine standards.

Amyloid beta peptide 1–42 (Aβ42) and 1–40 (Aβ40) were analyzed in supernatants of triple culture by ELISA Kit (Wuhan Fine Biotech Co., Wuhan, China) according to the manufacturer’s protocol.

CD63, CD81, and CD9 were quantified in the lysed EVs of hAFSCs by using ELISA assays, according to the manufacturer’s instructions (Cusabio Technology, Huston, TX, USA). In brief, EVs were lysed in a lysis buffer at a ratio of 1:3 (vol:vol) followed by three cycles of freeze and thaw. Protein concentration was found in all EV preparations, as analyzed by the Bradford test. Samples were run in duplicate. A standard curve was constructed using known concentrations of recombinant human standards.

### 4.13. Apoptosis Analysis

Apoptotic induction was determined at different DPI in differentiated SH-SY5Y and D54MG using a Guava^®^ Muse Cell Analyser (Luminex Corporation, Austin, TX, USA). Briefly, cells were trypsinized, washed, and stained with 7-AAD and Caspase 3/7 as described in the manufacturer’s protocol of The Muse^®^ Caspase 3/7 kit (Luminex Corporation, Austin, TX, USA). Results were shown as dot plot of Caspase 3/7 vs. Viability using Muse software version 1.3.

### 4.14. Western Blot Analysis

Cell extracts were obtained as previously described [60]. Briefly, cells were treated with lysis buffer (20 mM of Tris-Cl, pH 7.0; 1% Nonidet P-40; 150 mM of NaCl; 10% glycerol; 10 mM of EDTA; 20 mM of NaF; 5 mM of sodium pyrophosphate; and 1 mM of Na_3_VO_4_) and freshly added Sigma Aldrich Protease Inhibitor Cocktail and para-Nitrophenylphosphate (pNPP) at 4 °C for 20 min (all from Sigma Aldrich, St. Louis, MO, USA). Lysates were sonicated, cleared by centrifugation, and immediately boiled in SDS (Sigma Aldrich, St. Louis, MO, USA) reducing sample buffer.

Total lysates were loaded onto 4–16% SDS-PAGE. Primary antibodies, prepared as previously reported [56], were used against the following molecules: Tubulin (Sigma-Aldrich, St. Louis, MO, USA), APP (6E10, BioLegend, London, UK), CD86 (Novus Biologicals, Milano, Italy), gp91phox, p67phox, RAC1, SOD1 and SOD2 (Santa Cruz Biotechnology, CA, USA), LONP1 (from Cell Signaling Technology, Lieden, The Netherlands), Rab5 (HansaBioMed Life Sciences, Tallinn, Estonia), and β-Amyloid (BioLegend, London, UK). Secondary anti-bodies, used at 1:3000 dilution, were all from Thermo Fisher Scientific (Waltham, MA, USA).

### 4.15. Statistical Analysis

Experiments were performed in triplicate (biological replicates). For quantitative comparisons, values were reported as mean ± SD or SEM based on triplicate analysis (“*n*” refers to biological replicates) for each sample. To test the significance of observed differences among the study groups, one-way ANOVA with Bonferroni post hoc test or t-student test was applied. A *p*-value < 0.05 was considered statistically significant. Statistical analysis and plot layout were obtained by using GraphPad PrismR release 6.0 software.

## 5. Conclusions

In this study, we successfully developed a novel in vitro triple-culture model that effectively mimics the complex cellular environment and pathological hallmarks of mixed dementia. By integrating neurons, astrocytes, and microglia, this platform provided a robust framework to evaluate the therapeutic potential of Amniotic Fluid Stem Cell-derived Extracellular Vesicles (AFSC-EVs) against multifaceted neurodegenerative insults. Our findings demonstrate that AFSC-EVs exert a potent neuroprotective effect by targeting several key pillars of mixed dementia pathology. Notably, AFSC-EVs preserved neuronal mitochondrial integrity by promoting fusion and safeguarding morphological structure and function. While AFSC-EVs contribute exogenous antioxidant proteins to the system, our data reveal a significant reduction in SOD2 and FOXO3a mRNA levels in neurons. This suggests that EV treatment stabilizes the cellular environment to the point where further activation of endogenous antioxidant machinery is no longer required. Furthermore, the deep modulation of NOX2 subunits indicates that EVs act upstream to inhibit the generation of oxidative stress. The protective signature of AFSC-EVs was further confirmed by the prevention of ROS accumulation in macroglia and a decrease in IL6 production by microglia. Crucially, these molecular improvements translated into tangible phenotypic benefits, including the preservation of neurite morphology, increased cell viability, and a reduction in Aβ accumulation. In summary, this study highlights the multi-target therapeutic potential of AFSC-EVs as a promising cell-free strategy for the treatment of mixed dementia. By concurrently addressing oxidative damage, mitochondrial dysfunction, and neuroinflammation, these vesicles offer a comprehensive approach to halting the progression of complex cognitive decline.

## Figures and Tables

**Table 1:** Percentage of SH-SY5Y cell death in mono and triple culture (in co-culture with D54MG and HMC3). Analysis of SH-SY5Y death, treated with LPS or H_2_O_2_ or cotreated, was performed using Muse Cell Analyzer.

Treatment	Ctrl	LPS	H_2_O_2_	LPS + H_2_O_2_
SH-SY5Y mono	10 ± 1.2	18 ± 2.2	28 ± 2.1	25 ± 1.7
SH-SY5Y triple	11 ± 1.4	24 ± 1.8	22 ± 2.2	21 ± 1.6

## Data Availability

The original contributions presented in this study are included in the article/Supplementary Material. Further inquiries can be directed to the corresponding author(s).

## Supplementary Materials

The following supporting information can be downloaded at: https://www.mdpi.com/article/10.3390/ijms27114834/s1.
