# Supplementary figures and images for: Stem Cell-Derived Extracellular Vesicles Ameliorate the Neuron Mitochondrial Damage Induced by ROS-, LPS-Exposure: In Vitro Model of Neuron, Microglia, and Astrocyte Triple Co-Culture
Source: Int J Mol Sci. 2026 May 27;27(11):4834. doi: 10.3390/ijms27114834 (PMC13257201; doi:10.3390/ijms27114834)

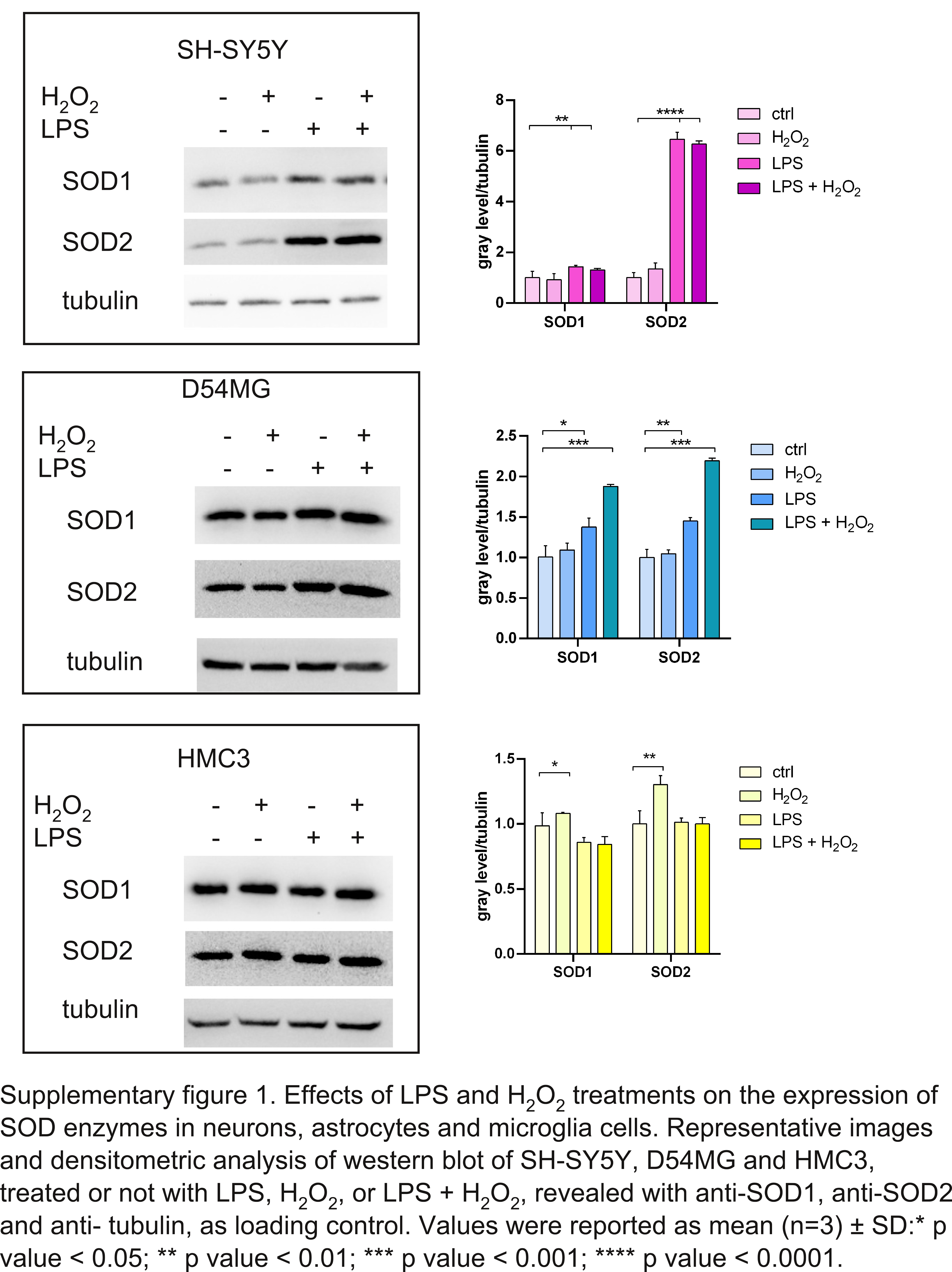

Supplement: Supplementary file 1 [file ijms-27-04834-s001.zip › ijms-4274369-supplementary.tif]
